# Cost-Effectiveness of Nivolumab Plus Cabozantinib Versus Sunitinib as a First-Line Treatment for Advanced Renal Cell Carcinoma in the United States

**DOI:** 10.3389/fphar.2021.736860

**Published:** 2021-12-13

**Authors:** SiNi Li, JianHe Li, LiuBao Peng, YaMin Li, XiaoMin Wan

**Affiliations:** ^1^ Clinical Nursing Teaching and Research Section, The Second Xiangya Hospital, Central South University, Changsha, China; ^2^ The Xiangya Nursing School, Central South University, Changsha, China; ^3^ School of Health and Related Research, Faculty of Medicine, Dentistry and Health, University of Sheffield, Sheffield, United Kingdom; ^4^ Department of Pharmacy, The Second Xiangya Hospital, Central South University, Changsha, China

**Keywords:** cost-effectiveness analyses, renal cell carcinoma, microsimulation, nivolumab, cabozantinib

## Abstract

**Background:** In a recent randomized, phase 3 trial (CheckMate 9ER), nivolumab combined with cabozantinib significantly improved patient outcomes compared with sunitinib. However, the cost-effectiveness of these novel agents for untreated advanced renal cell carcinoma (aRCC) remains unknown.

**Materials and Methods:** We constructed a microsimulation decision-analytic model to measure the healthcare costs and outcomes of nivolumab plus cabozantinib compared with those of sunitinib for patients with aRCC. The transition probability of patients was calculated from CheckMate 9ER using parametric survival modeling. Lifetime direct medical costs, life years (LYs), quality-adjusted life years (QALYs), and incremental cost-effectiveness ratios (ICERs) were estimated for nivolumab-plus-cabozantinib treatment compared with sunitinib from a US payer perspective. We conducted one-way and probabilistic sensitivity analyses and a series of scenario analyses to evaluate model uncertainty.

**Results:** Nivolumab plus cabozantinib was associated with an improvement of 0.59 LYs and 0.56 QALYs compared with sunitinib. However, incorporating nivolumab plus cabozantinib into first-line treatment was associated with significantly higher lifetime costs ($483,352.70 vs. $198,320.10), causing the incremental cost-effectiveness ratio for nivolumab plus cabozantinib to be $508,987/QALY. The patients’ age of treatment, first-line utility, and cost of nivolumab had the greatest influence on the model. The outcomes were robust when tested in sensitivity and scenario analyses.

**Conclusion:** For aRCC, substituting nivolumab plus cabozantinib in the first-line setting is unlikely to be cost-effective under the current willingness-to-pay threshold ($150,000/QALY). Significant price decreases for nivolumab used in first-line therapy would be needed to drop ICERs to a more diffusely acceptable value.

## Introduction

Renal cell carcinoma (RCC), the most common type of kidney cancer, was diagnosed in over 73,000 new cases and caused 14,000 deaths during 2020 in the United States (Choueiri et al., 2015; National Cancer Institute, 2021). Advanced RCC (aRCC) has the highest death rate among kidney cancers because this disease usually has no symptoms at the initial stage (Amzal et al., 2017). Delayed diagnosis leads to a large proportion (30%) of patients suffering from local advanced or metastatic disease and only an 11% 5-year relative survival rate (Fisher et al., 2013; Bhatt and Finelli, 2014; Sarfaty et al., 2018). Common symptoms of aRCC include pain, fatigue, anemia, anorexia, hypercalcemia, and venous thromboembolism (Cella, 2011). The financial burden of aRCC in the United States is considerable; previous studies have reported the annual cost of aRCC to be $107 to $556 million (Casciano et al., 2011).

Sunitinib, a vascular endothelial growth factor receptor (VEGFR) inhibitor, once regarded as a standard of care for the treatment of aRCC before 2018, has been replaced by novel immune checkpoint inhibitor (ICI) agents based on multiple respective randomized controlled trials (RCTs) (Motzer et al., 2018; Motzer et al., 2019; Powles et al., 2020). Both nivolumab (a programmed death 1 [PD-1] ICI antibody) and cabozantinib (a small-molecule inhibitor of tyrosine kinases) are approved agents for the treatment of aRCC and have been shown to enhance overall survival (OS) as single therapies in phase 3 trials (Motzer et al., 2015; Choueiri et al., 2017). Recently, a large randomized, open-label, phase 3 trial (the CheckMate 9ER trial) compared nivolumab combined with cabozantinib to standard sunitinib for patients with aRCC (Choueiri et al., 2021). This multicenter RCT was conducted in 125 medical centers of 18 countries. In this study, after a median follow-up period of 18.1 months, nivolumab plus cabozantinib showed a significant improvement in survival and quality of life (QoL) compared with sunitinib (Choueiri et al., 2021). The median progression-free survival (PFS) was 16.6 months in the nivolumab-plus-cabozantinib arm and 8.3 months in the sunitinib arm (hazard ratio (HR), 0.51; 95% confidence interval (CI), 0.41–0.64). The probability of 12-month OS with the nivolumab-plus-cabozantinib strategy and sunitinib strategy was 85.7 vs. 75.6% (HR for death, 0.60; 98.89% CI, 0.40–0.89) (Choueiri et al., 2021). The probability of adverse events (AEs) of any cause and AEs of grade 3 or higher during therapy in the nivolumab plus cabozantinib group was 99.7 and 75.3%, which was 0.6 and 4.7% higher than the sunitinib group’s, respectively (Choueiri et al., 2021).

Although incorporating the nivolumab-plus-cabozantinib strategy into the first-line setting has obviously increased health outcomes for aRCC patients, whether the substantial drug costs and adverse events (AEs) are justified by the health benefits gained remains unclear. Under the current healthcare setting, not only physicians but also policymakers and patients alike need plausible evidence as a framework to inform the value of novel combination strategies in oncology. Therefore, the aim of this study was to estimate the cost-effectiveness of nivolumab-plus-cabozantinib treatment compared with sunitinib as a first-line treatment for patients with aRCC from a US payer perspective.

## Methods

### Patients and Intervention

The baseline sample for our model was constructed to mirror the CheckMate 9ER trial (Choueiri et al., 2021). The mean age of the individual cohort was 62 years, and all patients had clear cell-type aRCC (Supplementary Table S1). Patients entered the model with untreated aRCC and received either nivolumab (240 mg every 2 weeks) plus cabozantinib (40 mg once daily) or standard sunitinib (50 mg once daily for 4 weeks of each 42-day cycle). After first-line failure, patients who experienced disease progression subsequently received axitinib (5 mg twice per day) and sorafenib (400 mg twice per day) as second-line and third-line treatments, respectively. This predefined treatment sequence was set based on NCCN clinical practice guidelines in oncology: kidney cancer, which listed currently available treatment of aRCC and recommended axitinib and sorafenib as subsequent treatment (National Comprehensive Cancer Network, 2021). All administration and dosage schedules for every line of treatment were obtained from the respective RCTs and are listed in Supplementary Table S2.

### Model Construction

**FIGURE 1:**
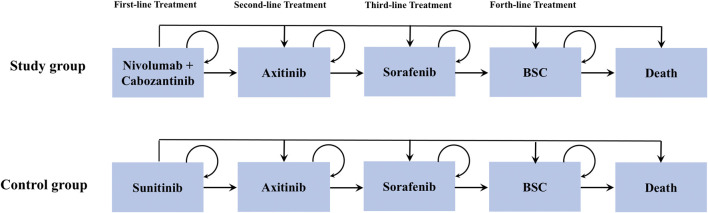
Model structure.

We created a microsimulation model to estimate the healthcare cost and clinical benefits associated with nivolumab plus cabozantinib versus sunitinib for patients with treatment-naïve aRCC using TreeAge Pro (TreeAge Software, Williamstown, MA). As illustrated in Figure 1, patients commenced treatment with a nivolumab-plus-cabozantinib or sunitinib followed by axitinib-sorafenib-best supportive care (BSC) treatment sequence until death. A 42-day model cycle was used to match the time interval with the CheckMate 9ERtrial and a lifetime horizon to assess direct healthcare costs and utilities related to each treatment arm (Choueiri et al., 2021). The primary outcomes of the model were used to estimate the additional cost for nivolumab-plus-cabozantinib treatment compared with sunitinib in 2021 US dollars for an incremental quality-adjusted life-year (QALY) yield (incremental cost-effectiveness ratio (ICER)). This study was performed from a US payer perspective with a willingness-to-pay (WTP) threshold of $150,000/QALY (Neumann et al., 2014), and both cost and utilities were discounted by 3% annually (Weinstein et al., 1996).

### Transition Probability

Patients transitioned between different health states based on transition probabilities calculated from the OS, PFS and discontinuation rate from multiple RCTs associated with the respective strategies (Rini et al., 2011; Motzer et al., 2014; Rini et al., 2020; Choueiri et al., 2021). First, the PFS survival curves from CheckMate 9 ER, AXIS, TIVO-3, and an RCT conducted by Robert J were used to estimate the probability of patients remaining in the PFS state of each treatment line by performing the standard extrapolation method designed by Guyot et al. (Guyot et al., 2012). Getdata Graph digitizer software was used to extract survival data points from survival curves to reconstruct pseudo-individual patient-level data (pseudo-IPD). Next, those pseudo-IPD data were fit to five standard parametric models (Weibull, exponential, lognormal, gamma, and log-logistic distributions), and the most appropriate distribution was selected for all curves based on the goodness of fit (Akaike information criterion) (Li et al., 2021). The PFS data of sorafenib in the two trials (Motzer et al., 2014; Rini et al., 2020) were pooled given the patient baseline characteristics and comparable trial eligibility criteria (Supplementary Table S1) between those two RCTs, similar to the analyses by Wu et al. (Wu and Shi, 2020). We used a log-logistic distribution to model survival, and all the survival parameters are listed in Table 1.

**TABLE 1:** Input parameters.

Parameters	Mean	Range	Distribution	Reference
Survival model of PFS in the full cohort				
Nivolumab + cabozantinib	Shape = 1.569; Scale = 15.064	—	Log-logistic	Choueiri et al. (2015)
Sunitinib	Shape = 1.646; Scale = 8.269	—	Log-logistic	Choueiri et al. (2015)
Axitinib	Shape = 1.4633; Scale = 6.6318	—	Log-logistic	National Cancer Institute, (2021)
Sorafenib	Shape = 2.281	—	Exponential	(Bhatt and Finelli, 2014; Amzal et al., 2017)
OS in the best support care	Shape = 1.613; Scale = 13.857	—	Log-logistic	Sarfaty et al. (2018)
Probability of treatment discontinuation as a result of AE (%)				
Nivolumab + cabozantinib	19.7	—	Beta	Fisher et al. (2013)
Sunitinib	16.9	—	Beta	Fisher et al. (2013)
Axitinib	8.49	—	Beta	National Cancer Institute, (2021)
Sorafenib	18.11	—	Beta	(Bhatt and Finelli, 2014; Amzal et al., 2017)
Probability of treatment mortality as a result of AE (%)				
Nivolumab + cabozantinib	0.31	—	Beta	Choueiri et al. (2015)
Sunitinib	0.61	—	Beta	Choueiri et al. (2015)
Axitinib	0	—	—	National Cancer Institute, (2021)
Sorafenib	0.7	—	Beta	(Bhatt and Finelli, 2014; Amzal et al., 2017)
Probability of background death	—	—	—	Cella, (2011)
Drug cost				
Nivolumab 240 mg	6,849.84	5,479.87–8,219.81	Gamma	Casciano et al. (2011)
Cabozantinib 60 mg	491.30	393.04–589.56	Gamma	Motzer et al. (2018)
Sunitinib 50 mg	623.08	498.46–747.70	Gamma	Motzer et al. (2018)
Axitinib 5 mg	265.05	212.04–318.06	Gamma	Powles et al. (2020)
Sorafenib 200 mg	174	139.20–208.80	Gamma	Motzer et al. (2019)
Cost of best support care	1,256	1,022–1,489	Gamma	Motzer et al. (2018)
Management of AEs				
Nivolumab + cabozantinib	1,214.68	971.74–1,457.61	Gamma	(Choueiri et al., 2015; Choueiri et al., 2017)
Sunitinib	6,632.78	5,306.22–7,959.34	Gamma	(Choueiri et al., 2015; Choueiri et al., 2017)
Axitinib	4,660.34	3,728.27–5,592.41	Gamma	(Motzer et al., 2015; Motzer et al., 2018; Choueiri et al., 2021; National Cancer Institute, 2021)
Sorafenib	2,284.81	556.72–835.08	Gamma	(Bhatt and Finelli, 2014; Amzal et al., 2017; Choueiri et al., 2021)
Administration cost				
IV infusion, single or initial drug (≤1 h)	148.3	118.64–177.93	Gamma	National Comprehensive Cancer Network, (2021)
Utilities				
First-line treatment	0.82	0.65–0.98	Beta	Choueiri et al. (2017)
Second-line treatment	0.77 (SD: 0.24)	0.616–0.924	Beta	Neumann et al. (2014)
Third-line treatment	0.66 (SD: 0.30)	0.528–0.792	Beta	Weinstein et al. (1996)
Fourth-line treatment, BSC	0.494	0.403–0.570	Beta	Rini et al. (2011)
Disutility due to AEs (grade ≥3)	0.157	0.11–0.204	Beta	Rini et al. (2020)
Average patient weight (kg)	70	49.0–93.8	Beta	Powles et al. (2020)

OS, overall survival; PFS, progression-free survival; AE, adverse event.

Second, we also took the discontinuation rate associated with AEs into account, with transition probabilities collected from the literature (Rini et al., 2011; Motzer et al., 2014; Rini et al., 2020; Choueiri et al., 2021). Finally, the probability of transitioning to death of each model cycle was defined as the value of combining data concerning treatment-related serious AEs from respective RCTs with an age-specified background mortality rate from the 2019 US Life Table (Arias and Xu, 2019) and observed mortality rate using survival data from each trial (Rini et al., 2011; Motzer et al., 2014; Rini et al., 2020; Choueiri et al., 2021). The probability of death from the BSC phase was calculated on the basis of the OS curve of the RECORD-1 trial using the same approach with the transition probabilities of PFS (Motzer et al., 2008). Baseline evaluations of clinical transition probabilities are displayed in Table 1.

### Costs and Utilities

Only direct costs were adopted as follows: drug acquisition costs, administration cost, management of AEs, and BSC. The unit prices of nivolumab in the United States were derived from the Centers for Medicare and Medicaid Services (CMS) on the basis of the 2021 average sale price (ASP Drug Pricing Files, 2021). The costs of oral drugs (sunitinib, cabozantinib, axitinib, and sorafenib) not included in CMS were collected from public literature and databases. (ASP Drug Pricing Files, 2021; Su et al., 2021; Lu et al., 2020; Watson et al., 2020). Medication costs were estimated using a baseline patient with a weight of 70 kg since weight loss effects in disease were considered (Wan et al., 2019; Lu et al., 2020). The overall costs associated with the management of grade 3 or 4 AEs and BSC were derived from the previous literature (Perrin et al., 2015; Wan et al., 2019; Lu et al., 2020; Agency for Healthcare Research and Quality, US Dept of Health and Human Services, 2021). The drug infusion cost was obtained from the 2021 CMS Physician Fee Schedule, with the duration of drug administration based on the CheckMate 9ER trial (Centers for Medicare and Medicaid Services, 2021).

The health utility scores, which range from 0 (death) to 1 (perfect health), reflect the value of QoL in a particular health state. Based on previously published studies, we set the utility values of first-, second-, and third-line treatments and the BSC phases to 0.82, 0.77, 0.66, and 0.494, respectively (Cella et al., 2018; de Groot et al., 2018; Wan et al., 2019; Patel et al., 2021a). We also considered the utility decrement (−0.157) due to AEs (Wu et al., 2018). QALYs were estimated by multiplying the time duration in a specific state by the utility value related to that state.

### Sensitivity Analysis

To test the uncertainty in evaluating input parameters and to assess model robustness, a series of sensitivity analyses, including one-way sensitivity analyses and probabilistic sensitivity analyses (PSAs), were performed. In accordance with established methods, costs were changed by 20% from their baseline values, and the upper and lower bounds were varied over the 95% CI of variables for those parameters with CIs such as utilities (Kohn et al., 2017; Zhang et al., 2012; Goulart and Ramsey, 2011). In the one-way sensitivity analyses, the value of one variable at a time was changed within a predefined range to explore the individual impact of each variable on ICERs for all parameters in Table 1. In PSA, we performed a Monte Carlo simulation of 5,000 iterations of 2000 patients to account for the change in all input parameters at once. All parameters were randomly sampled from the specific distributions. According to recommended distributions in accordance with previous cost-effectiveness analysis, we assumed a gamma distribution for costs, a beta distribution for utility values and incidence of AEs, and a normal distribution for both the weight and starting age of patients (Kohn et al., 2017; Patel et al., 2021b). Based on the PSA, a cost-effectiveness acceptability curve was obtained and used to illustrate the probability that the two treatment strategies could be regarded as the most cost-effective under different WTP thresholds.

We also included three scenario analyses in this study. In the first scenario analysis, we varied the nivolumab to 75, 50, and 25% of its original price. In the second, the time horizon was changed to 5, 10, and 15 years to evaluate the impact of the OS and PFS extrapolations used in the model. In the final scenario analysis, we set patients who would experience a certain proportion (18.9% in the nivolumab-plus-cabozantinib arm and 32.9% in the sunitinib arm) switching to the BSC phase after disease progression from first-line therapy, in accordance with the CheckMate 9ER and AXIS trials.

## Results

### Base Case Analysis

The nivolumab-plus-cabozantinib treatment strategy was associated with an improvement of 0.56 QALYs and 0.59 LYs compared with sunitinib (2.97 vs. 2.41 QALYs and 3.9 vs. 3.31 LYs, respectively). However, the nivolumab-plus-cabozantinib strategy was associated with dramatically greater healthcare costs ($483,352.70 vs. $198,320.10, respectively), with an additional cost of $285,033. The ICER of nivolumab plus cabozantinib as a first-line treatment was $508,987/QALY compared with standard sunitinib (Table 2).

**TABLE 2:** Base case results.

Results	Nivolumab + cabozantinib	Sunitinib	ICER
Total cost of regimen, $	483,352.7	198,320.1	—
Life-years	3.90	3.31	—
QALYs	2.97	2.41	—
Per LY	—	—	483,106
Per QALY	—	—	508,987

ICER, incremental cost-effectiveness ratio; LY, life year; QALYs, quality-adjusted life years.

### Sensitivity Analyses

**FIGURE 2:**
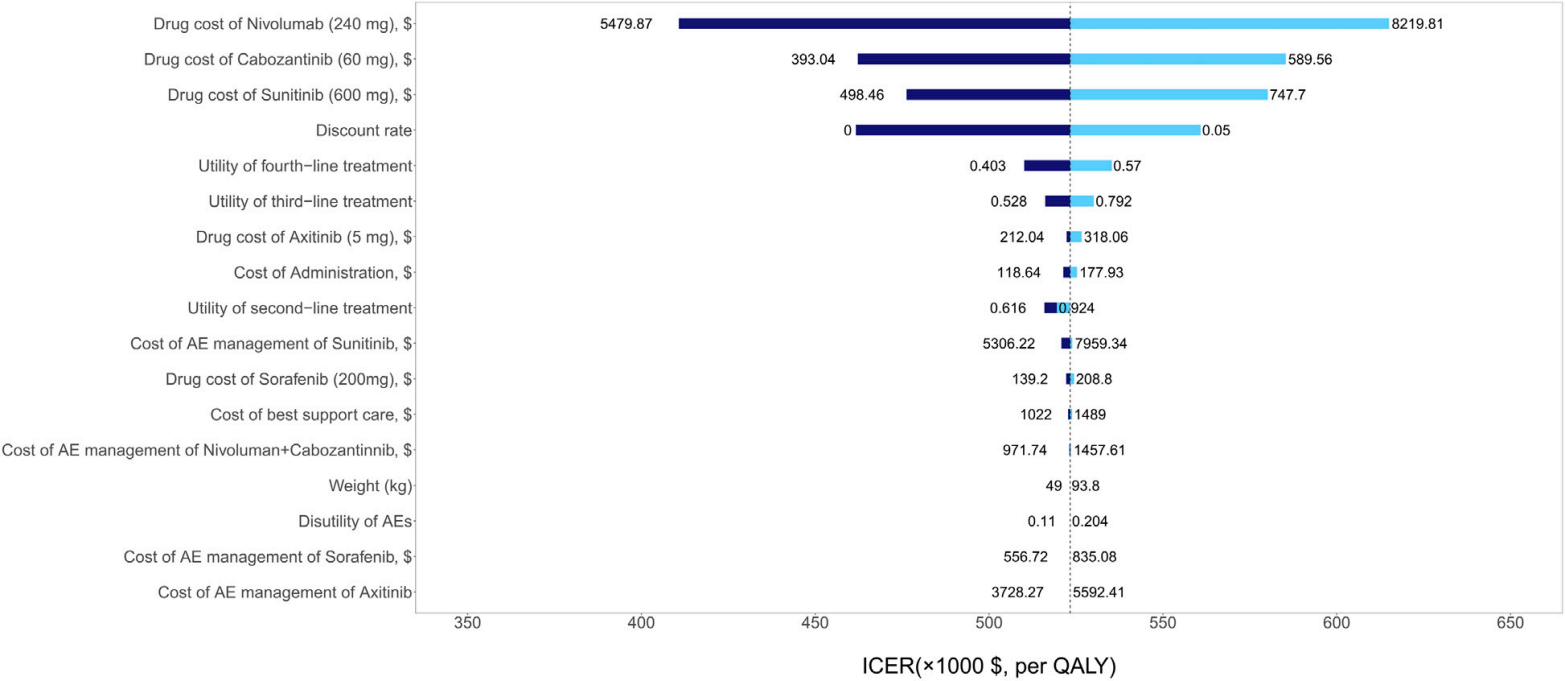
Tornado diagrams showing the effect of lower and upper values of each parameter on the ICERs of the nivolumab-plus-cabozantinib versus sunitinib strategy.

**FIGURE 3:**
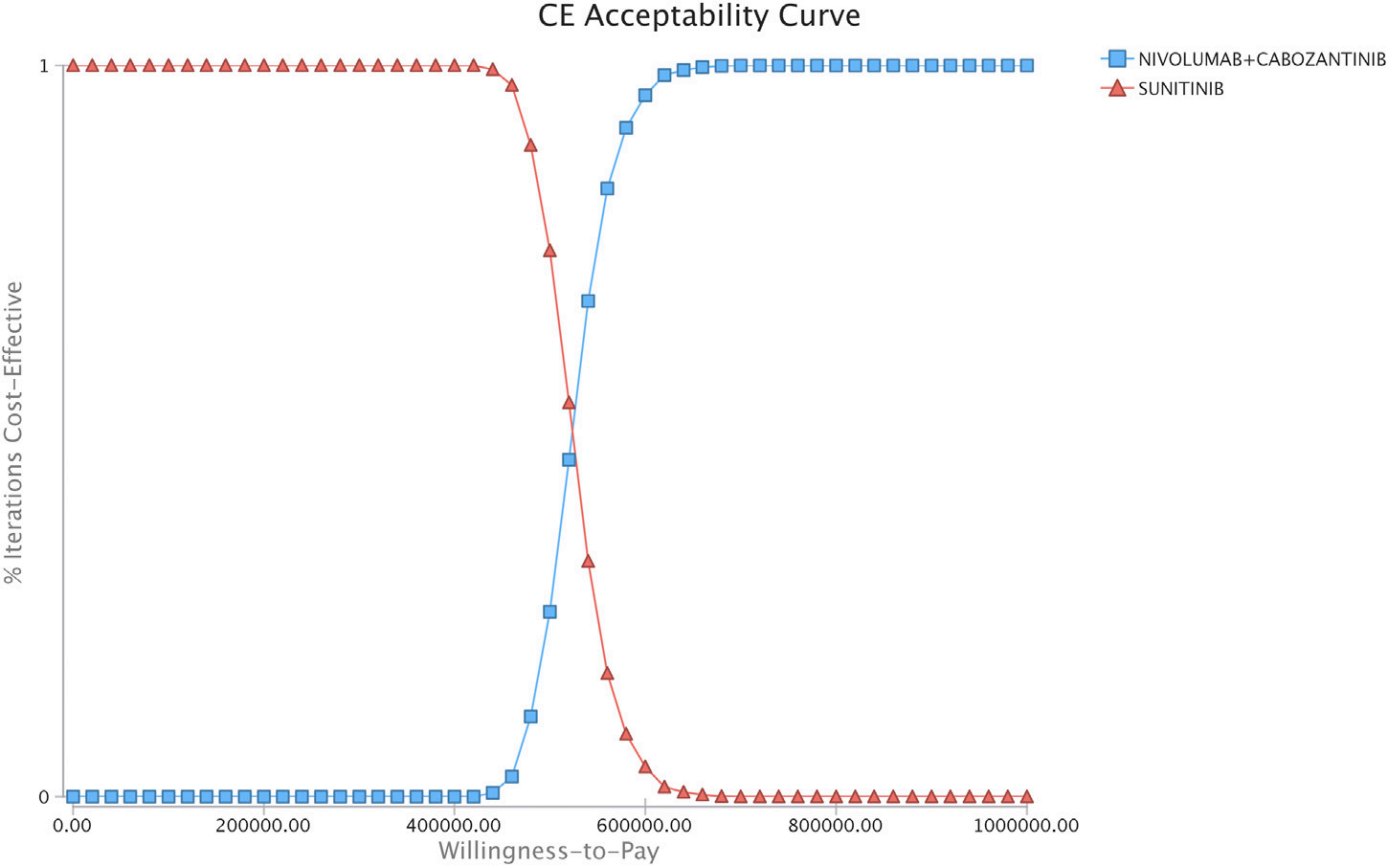
Acceptability curves comparing the cost-effectiveness of nivolumab plus cabozantinib vs. sunitinib strategies for patients with advanced renal cell carcinoma. CE, cost effectiveness..

The one-way sensitivity analyses showed that patients’ age of starting treatment, the utility of first-line therapy, and the drug cost of nivolumab had a considerable influence on model outcomes. Other variables, such as the drug costs of axitinib and sorafenib, the utility of third- and fourth-line therapy, and weight, had a moderate impact on our estimated ICER (Figure 2). The PSA results showed that there was a 100% probability of the nivolumab-plus-cabozantinib strategy being regarded as not cost-effective at the WTP threshold of $150,000/QALY compared with sunitinib (Figure 3).

### Scenario Analyses

The first scenario analyses revealed that reductions in the drug price of nivolumab prescribed in the first-line therapy of 75, 50, and 25% would lead to lower ICERs of $410,584/QALY, $295,405/QALY, and $177,747/QALY, respectively. However, it is still unlikely to be cost-effective under the current WTP threshold. The second scenario analyses indicated that the ICERs were $929,570/QALY, $603,897/QALY, and $547,448/QALY when we adjusted the time horizon to 5, 10, and 15 years, respectively. In the final scenario analyses, a certain percentage of patients turned to the BSC phase after progressing from first-line treatment rather than receiving second-line treatment. However, the results found that the model outcome did not vary significantly due to this adjustment, with an ICER of the nivolumab-plus-cabozantinib versus sunitinib strategy of $332,839/QALY. We list all the results of scenario analyses in Supplementary Table S4.

## Discussion

The recent CheckMate 9ER trial reported that the combination of nivolumab and cabozantinib could improve PFS and OS compared with standard sunitinib. By incorporating findings from this trial, we developed a microsimulation model to evaluate the cost-effectiveness of nivolumab plus cabozantinib as a first-line treatment. Under current drug prices in the US, where nivolumab costs in excess of $14,000 per month, first-line nivolumab plus cabozantinib was not cost-effective when compared with sunitinib, with an ICER of $508,987/QALY. The one-way sensitivity analyses indicated that the patients’ age of starting treatment had the greatest influence on the model. Lowering the baseline starting treatment age to 18 allowed patients to have more treatment time and more potential opportunities to accrue incremental benefit from delayed disease progression. The PSA showed that the probability of nivolumab plus cabozantinib being cost-effective was 0% in the first-line settings for a WTP of $150,000/QALY. This uncertainty analysis reveals a high likelihood that the nivolumab-plus-cabozantinib strategy exceeds the usually accepted and reasonable values for cost-effective incremental costs of care. Although we performed a series of scenario analyses in this study, the results appeared to accord with base case analyses, and the outcomes did not significantly change.

Although the result of this study revealed that it is unlikely for the nivolumab-plus-cabozantinib strategy to be cost-effective compared with sunitinib under the current WTP threshold of the US, the nivolumab-plus-cabozantinib strategy still has a considerable value in clinical practice due to its significant clinical efficacy. However, the high price of anti-cancer drugs might result in a certain risk for financial toxicity for patients with aRCC. Patients who cannot undertake the significant financial burden of the out-of-pocket fee will suffer from financial toxicity, leading to delay, discontinuity, and abandonment of treatment among patients diagnosed with severe cancer. Therefore, the healthcare system needs to ensure that novel and efficacy treatment strategies could be accessible and affordable for patients and minimize its financial burden. To better understand this, we further performed scenario four analyses that adjusted the price of nivolumab + cabozantinib strategy to 75, 50, and 25% of its original price to inform the policymaker. Moreover, the results of scenario four demonstrated that if the price of nivolumab + cabozantinib strategy decreased 50 and 25% of its original price, the ICER will drop to $107004/QALY and -$7,584/QALY, which could be considered as a cost-effective and very cost-effectiveness strategy, respectively, compared with sunitinib.

This study has several highlights. First, our model was performed on the basis of results from a multicenter, randomized, phase 3 clinical trial directly comparing nivolumab plus cabozantinib with sunitinib in the first-line setting. Second, to our knowledge, this is the first cost-effectiveness study of the nivolumab-plus-cabozantinib strategy in the first-line setting for patients with aRCC. Third, we took AEs into consideration, such as treatment discontinuation due to AEs, along with costs and disutility associated with drug toxicity. Fourth, we conducted multiple scenario analyses to reflect clinical practice in the real world; for example, some patients experienced discontinuation of treatment and switched to the BSC phase due to other causes. Finally, a microsimulation model was adopted to explain the heterogeneity of patients in our study.

This study also had some weaknesses that merit discussion. First, this study was performed from a US payer perspective, and the results of this study could not be applied in other countries because of the diversity in the costs, medical policy, and healthcare systems among different countries. Second, although we collected the value of utilities from the published aRCC cost-effectiveness analyses, it could not precisely reflect the population simulated in the model. The accuracy of the outcomes will improve if the evaluated utilities for patients with aRCC who receive nivolumab plus cabozantinib as first-line treatment are available in the future. Third, we did not consider the monotherapy of nivolumab or cabozantinib in this study due to a lack of head-to-head trials. Finally, we did not take a societal perspective into account because of the barrier related to obtaining the costs and benefits across patients and different sectors together, including healthcare costs associated with both informal and non-health sectors.

## Conclusion

In summary, for aRCC patients, the first-line treatment of the nivolumab-plus-cabozantinib strategy could not be considered a cost-effective strategy at the current WTP threshold of $150,000 in the United States compared with sunitinib.

## Data Availability

The original contributions presented in the study are included in the article/Supplementary Material; further inquiries can be directed to the corresponding authors.
