# Supplementary material for: Cost-Effectiveness of Nivolumab Plus Cabozantinib Versus Sunitinib as a First-Line Treatment for Advanced Renal Cell Carcinoma in the United States
Source: Front Pharmacol. 2021 Dec 13;12:736860. doi: 10.3389/fphar.2021.736860 (PMC8711761; doi:10.3389/fphar.2021.736860)

**eTable 1. Summary table of included studies.**

|  | Key characteristic | | | | | | | | | |
| --- | --- | --- | --- | --- | --- | --- | --- | --- | --- | --- |
| Study | Study design | Treatment | Sample size | Type | Prior therapies | Age (yrs) | Male (%) | IMDC prognostic risk score | | |
|  |  |  |  |  |  |  |  | F | I | P |
| First-line treatment | | | | | | | | | | |
| CheckMate 9ER | Phase III open-label RCT | NIV+ Cabo  Sunitinib | 323  328 | CC | None | 62  61 | 77.1  70.7 | 22.9  22.0 | 58.2  57.3 | 18.9  20.7 |
| Second-line treatment | | | | | | | | | | |
| AXIS | Phase III open-label RCT | Axitinib  sorafenib | 361  362 | CC | 1 prior first-line treatment | 61  61 | 73  71 | NA | | |
| Third-line treatment | | | | | | | | | | |
| Robert J Motzer et al., 2014 | Phase III RCT | Dovitinib  Sorafenib | 280  284 | CC | 1 prior TKI plus 1 prior mTORi | 61  62 | 75  77 | NA | | |
| TIVO-3 | Phase III open-label RCT | Tivozanib  Sorafenib | 173  170 | CC | 2-3 prior systemic  regimens | 62  63 | 72  73 | 19  21 | 62  60 | 18  19 |

*F = Favorable; I = Intermediate; P = Poor; IMDC = International Metastatic Renal-Cell Carcinoma Database Consortium; CC = Clear cell.

**eTable 2. Drug dose and costs**

| Drug | Dose | Route | Unit Price ($) | Cost for 1 model cycle ($, 42 days) |
| --- | --- | --- | --- | --- |
| Nivolumab | 240 mg every 2 weeks | IV | 28.541/mg | 20549.52 |
| Cabozantinib | 40 mg/d | oral | 8.1883/mg | 13756.344 |
| Sunitinib | 50 mg/d for 4 weeks followed by 2 weeks off treatment | oral | 12.4616/mg | 17446.24 |
| Axitinib | 5mg twice/d | oral | 53.01/mg | 5566.05 |
| Sorafenib | 400mg twice/d | oral | 0.87/mg | 7308 |

**eTable 3. Background mortality rate**

Estimates of background mortality rate for each age are provided in the US life table; Arias E, Heron M, Xu J. United States Life Tables, 2019. Natl Vital Stat Rep. 2019; 68:1-65.

| Age (years) | Background | Age (years) | Background | Age (years) | Background |
| --- | --- | --- | --- | --- | --- |
| 18 | 0.000603 | 54 | 0.007003 | 90 | 0.166829 |
| 19 | 0.000698 | 55 | 0.007607 | 91 | 0.185047 |
| 20 | 0.000795 | 56 | 0.008219 | 92 | 0.204441 |
| 21 | 0.000889 | 57 | 0.008857 | 93 | 0.224919 |
| 22 | 0.000970 | 58 | 0.009542 | 94 | 0.246354 |
| 23 | 0.001424 | 59 | 0.010285 | 95 | 0.26890 |
| 24 | 0.001497 | 60 | 0.011098 | 96 | 0.291442 |
| 25 | 0.001561 | 61 | 0.011952 | 97 | 0.314700 |
| 26 | 0.001624 | 62 | 0.012814 | 98 | 0.338142 |
| 27 | 0.001682 | 63 | 0.013657 | 99 | 0.361537 |
| 28 | 0.001737 | 64 | 0.014502 | 100 | 1 |
| 29 | 0.001792 | 65 | 0.015384 |  |  |
| 30 | 0.001847 | 66 | 0.016444 |  |  |
| 31 | 0.001900 | 67 | 0.017624 |  |  |
| 32 | 0.001952 | 68 | 0.018968 |  |  |
| 33 | 0.002003 | 69 | 0.029586 |  |  |
| 34 | 0.002053 | 70 | 0.022109 |  |  |
| 35 | 0.002111 | 71 | 0.024359 |  |  |
| 36 | 0.002174 | 72 | 0.026347 |  |  |
| 37 | 0.002233 | 73 | 0.028810 |  |  |
| 38 | 0.002285 | 74 | 0.031309 |  |  |
| 39 | 0.002340 | 75 | 0.034486 |  |  |
| 40 | 0.002413 | 76 | 0.038026 |  |  |
| 41 | 0.002516 | 77 | 0.042286 |  |  |
| 42 | 0.002649 | 78 | 0.046547 |  |  |
| 43 | 0.002811 | 79 | 0.051534 |  |  |
| 44 | 0.002999 | 80 | 0.057008 |  |  |
| 45 | 0.003203 | 81 | 0.062923 |  |  |
| 46 | 0.003433 | 82 | 0.069911 |  |  |
| 47 | 0.003709 | 83 | 0.078099 |  |  |
| 48 | 0.004047 | 84 | 0.086754 |  |  |
| 49 | 0.004445 | 85 | 0.096549 |  |  |
| 50 | 0.004874 | 86 | 0.106472 |  |  |
| 51 | 0.005331 | 87 | 0.119677 |  |  |
| 52 | 0.005844 | 88 | 0.134128 |  |  |
| 53 | 0.006408 | 89 | 0.149846 |  |  |

eTable 4. The results of scenario analyses.

| Strategy | Total cost | LY | QALY | ICER |
| --- | --- | --- | --- | --- |
| Scenario 1 | | | | |
| Adjust nivolumab 75% of its original price in the first-line setting. | | | | |
| Nivolumab + Cabozantinib | 419436 | 3.93 | 2.94 | 410584 |
| Sunitinib | 199450 | 3.34 | 2.40 | - |
| Adjust nivolumab 50% of its original price in the first-line setting. | | | | |
| Nivolumab + Cabozantinib | 361998 | 3.93 | 2.97 | 295405 |
| Sunitinib | 200080 | 3.35 | 2.43 | - |
| Adjust nivolumab 25% of its original price in the first-line setting. | | | | |
| Nivolumab + Cabozantinib | 297240 | 3.95 | 2.96 | 177747 |
| Sunitinib | 199465 | 3.34 | 2.41 | - |
| Scenario 2 | | | | |
| 5 years | | | | |
| Nivolumab + Cabozantinib | 447326 | 2.96 | 2.24 | 929570 |
| Sunitinib | 186327 | 2.70 | 1.96 | - |
| 10 years | | | | |
| Nivolumab + Cabozantinib | 477585 | 3.67 | 2.74 | 603897 |
| Sunitinib | 196161 | 3.20 | 2.27 | - |
| 15 years | | | | |
| Nivolumab + Cabozantinib | 485649 | 3.85 | 2.89 | 547448 |
| Sunitinib | 198710 | 3.28 | 2.37 | - |
| Scenario 3 | | | | |
| 18.9% in nivolumab-plus-cabozantinib arm and 32.9% in sunitinib arm of patents switch to BSC | | | | |
| Nivolumab + Cabozantinib | 477440 | 3.73 | 2.81 | 332839 |
| Sunitinib | 169017 | 2.71 | 1.88 | - |
| Scenario 4 | | | | |
| Adjust Nivolumab + Cabozantinib 75% of its original price in the first-line setting | | | | |
| Nivolumab + Cabozantinib | 374961 | 3.75 | 2.83 | 220266 |
| Sunitinib | 170663 | 2.72 | 1.90 | - |
| Adjust Nivolumab + Cabozantinib 50% of its original price in the first-line setting | | | | |
| Nivolumab + Cabozantinib | 268152 | 3.75 | 2.82 | 107004 |
| Sunitinib | 169935 | 2.72 | 1.90 | - |
| Adjust Nivolumab + Cabozantinib 50% of its original price in the first-line setting | | | | |
| Nivolumab + Cabozantinib | 163308 | 3.76 | 2.82 | -7584 |
| Sunitinib | 170258 | 2.73 | 1.91 | - |

eFigure 1: Parametric Distributions for First-Line Treatment.

*KM = Kaplan-Meier; NIV+CABO =Nivolumab + Cabozantinib;


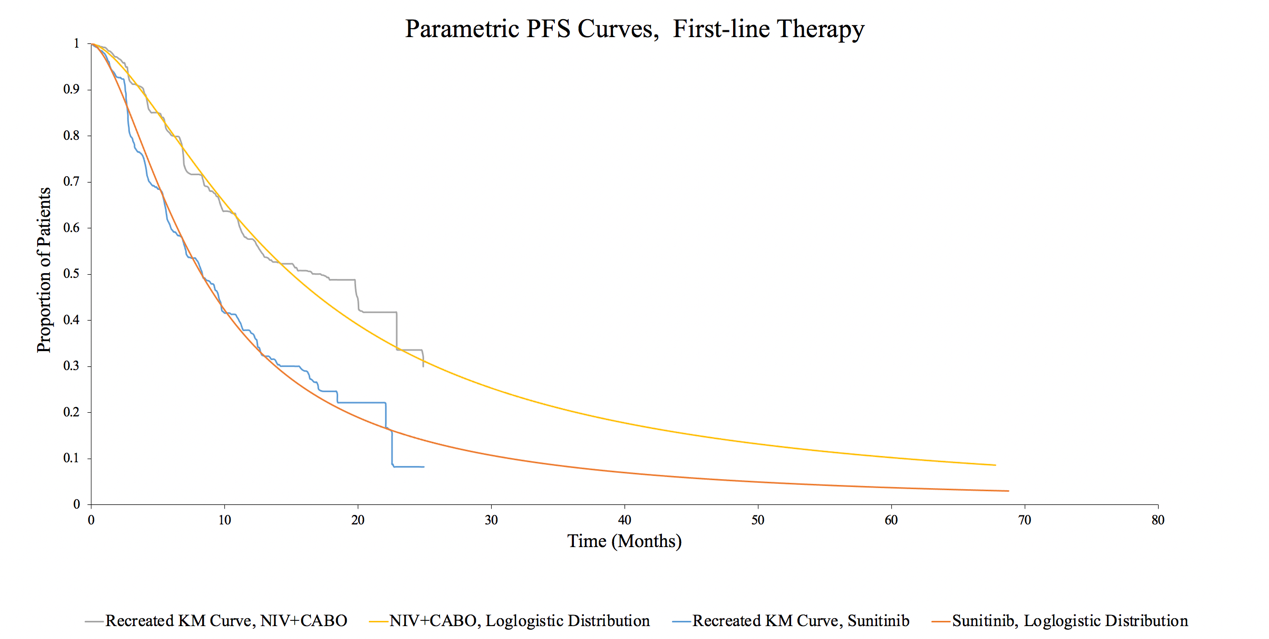


eFigure 2: Parametric Distributions for Second-Line Treatment. KM = Kaplan-Meier.


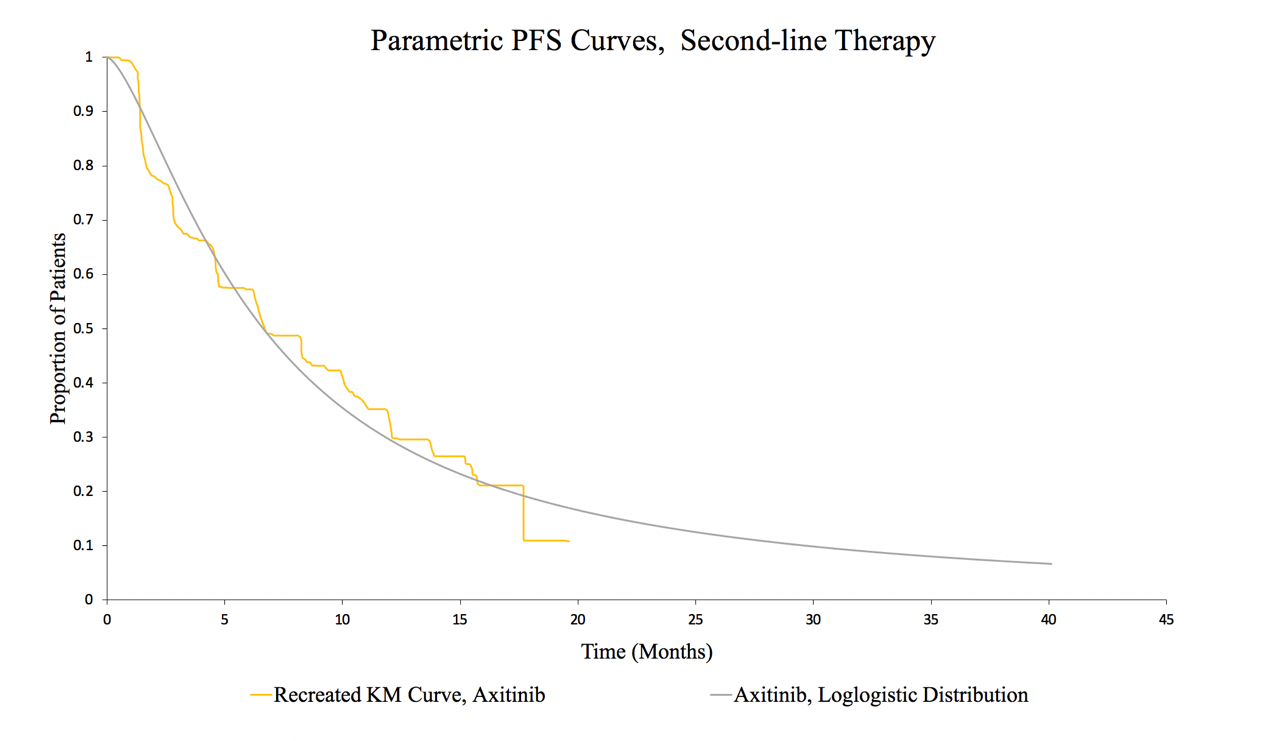


eFigure 3: Parametric Distributions for Third-Line Treatment. KM = Kaplan-Meier.


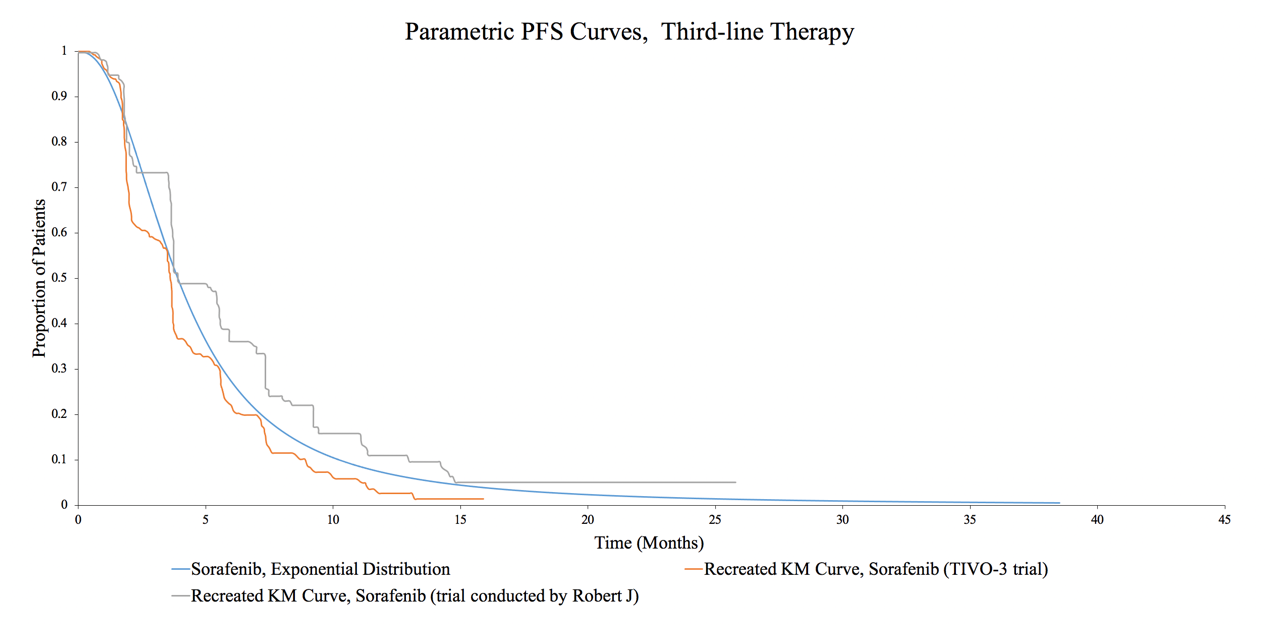


eFigure 4: Parametric Distributions for Best Support Care State.

*BSC = Best support care.


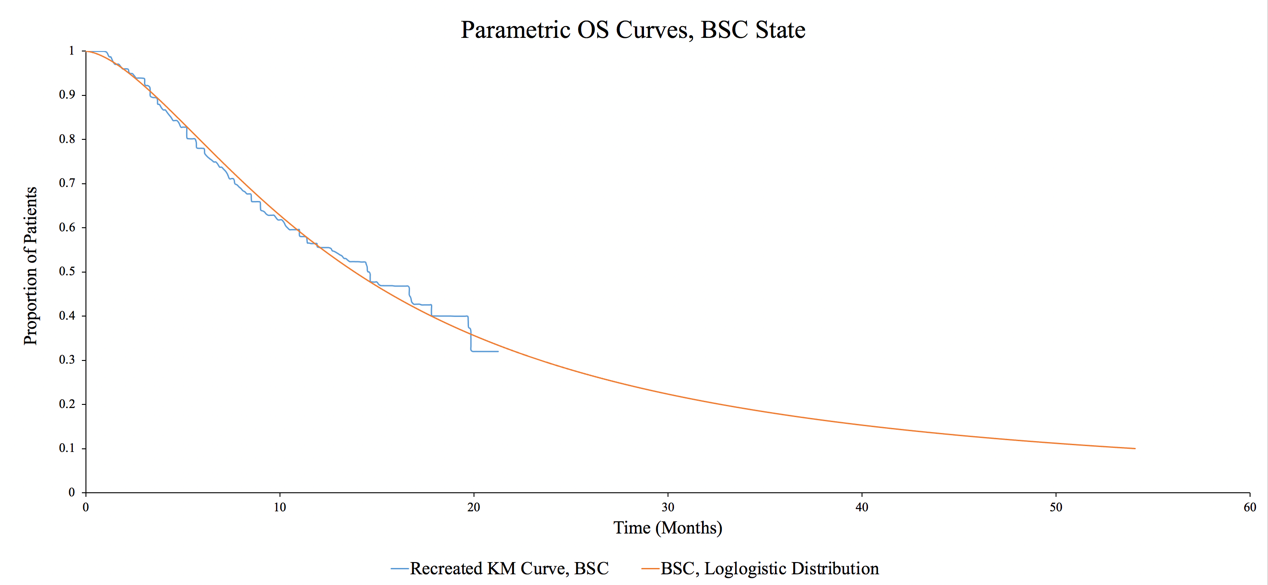

Supplement: Supplementary file 1 [file DataSheet1.DOCX]
